# Improving image quality in terbium‐161 phantom imaging: Quantitative evaluation of DEW and TEW scatter correction methods

**DOI:** 10.1002/acm2.70672

**Published:** 2026-06-25

**Authors:** Melek Can, Gamze Çapa Kaya, Gül Gümüşer, Yasemin Parlak

**Affiliations:** ^1^ Program of Nuclear Medicine Techniques Vocational School of Health Services Dokuz Eylul University Izmir Turkey; ^2^ Department of Medical Physics Institute of Health Sciences Dokuz Eylul University Izmir Turkey; ^3^ Department of Nuclear Medicine Faculty of Medicine Dokuz Eylul University Izmir Turkey; ^4^ Department of Nuclear Medicine Faculty of Medicine Manisa Celal Bayar University Manisa Turkey

**Keywords:** contrast to noise ratio, scatter correction, signal to noise ratio, SPECT, Tb‐161

## Abstract

**Background:**

Terbium‐161 (Tb‐161) emits gamma rays and beta radiation, enabling both therapeutic and imaging applications. However, the multiple gamma emissions of ^161^Tb can affect image quality by increasing the scattering rate during SPECT imaging. To improve image quality, appropriate scatter correction methods, such as Dual‐Energy Window (DEW) and Triple‐Energy Window (TEW) need to be optimized. Although these methods are used in clinical practice, studies investigating the efficacy of spectral analysis approaches for next‐generation radionuclides with multiple gamma emissions, such as ^161^Tb, are limited.

**Purpose:**

This study aims to evaluate the effects of DEW and TEW scatter correction methods on image quality and quantitative accuracy in ^161^Tb SPECT imaging compared to uncorrected images.

**Methods:**

Three distinct reference geometries were utilized to determine the gamma camera calibration factor (CF) and to evaluate the image quality parameters. Five image protocols were used, each with different combinations of main photopeak and scatter energy windows. Image quality parameters (CF, contrast‐to‐noise ratio (CNR), signal‐to‐noise ratio (SNR), spatial resolution) and activity bias (%) were compared using LifeX software in both uncorrected and corrected SPECT images.

**Results:**

The CF calculated in images acquired with an energy window of 48.9 keV was found to be higher than that calculated in an energy window of 74.6 keV. The best CNR was calculated for images acquired in air with a photopeak of 48.9 keV ± 10% and a scatter window of 6%. In scattered media, it was observed in images obtained with a photopeak of 74.6 keV ± 10% and a scatter window of 6%. When scatter correction was applied, the images’ SNR values decreased slightly, ranging from 1.188 to 1.724 across all media. Scatter correction techniques reduced the FWHM values for all protocols except the air medium, thereby improving spatial resolution. Activity bias results showed an overestimation in uncorrected protocols including the 48.9 keV peak. Conversely, utilizing the 74.6 keV peak with TEW correction improved quantitative accuracy to a 3.7%–4.2% absolute bias.

**Conclusion:**

Our results show that both DEW and TEW correction approaches improve spatial resolution and increase CNR by reducing scattering contributions and background noise. However, as expected with the subtraction of scattered effects, these enhancements are accompanied by a slight decrease in SNR. The TEW method performed better than the DEW method in terms of quantitative accuracy under scattering conditions. In SPECT imaging using therapeutic amounts of Tb‐161, high image quality can be achieved with an energy window of 74.6 keV ± 10%.

## 1 INTRODUCTION

Radionuclide therapy (RNT) is a treatment method that aims to selectively deliver high doses of radiation to tumor cells using pharmaceutical agents labeled with radioactive isotopes that emit beta or alpha particles, while minimizing the impact on healthy tissues. RNT is an important part of personalized therapy due to its biotargeting capacity.^1^, ^2^ In recent years, RNTs have been increasingly used in malignancies, such as neuroendocrine tumors and metastatic castration‐resistant prostate cancer.^3^ Peptide receptor radionuclide therapy (PRRT) and prostate‐specific membrane antigen (PSMA)‐targeted therapies have demonstrated promising results in patients refractory to standard treatments.^4^ Nevertheless, Lutetium‐177 (^177^Lu), which is frequently employed in RNT applications, can demonstrate suboptimal efficacy in tumors smaller than 5 mm due to its average tissue penetration of 2 mm.^5^, ^6^ As a result, this limitation may compromise treatment efficacy, especially in early‐stage or minimal residual disease scenarios.

To overcome these limitations, Terbium‐161 (^161^Tb), which has similar physical and chemical properties to ^177^Lu, has emerged as a promising alternative. Tb‐161 is a next‐generation radionuclide that emits gamma rays and beta radiation, enabling both therapeutic and imaging applications.^7^ With a physical half‐life of 6.89 days, this isotope has a pharmacokinetic profile similar to that of ^177^Lu and can be easily radiolabeled with the same chelator methods. Tb‐161 emits beta particles with a mean energy of 154.3 keV and gamma rays at 74.6 keV (10.3%) and 48.9 keV (17%), which are suitable for imaging. Furthermore, it emits Auger electrons (8.94 keV per decay) and internal conversion electrons (mean 39.38 keV), which can yield a substantially higher biological effectiveness.^6^, ^8^, ^9^, ^10^

The main radiobiological advantage of ^161^Tb is attributed to its numerous decay channels. While beta particles exert cytotoxic effects over a range of a few millimeters, Auger and internal conversion electrons act at nanometer and micrometer dimensions, releasing energy over much shorter distances, thereby creating intense ionization within the cell. This mechanism effectively drives cancer cells into apoptosis by increasing the frequency of intracellular DNA double‐strand breaks.^5^, ^8^, ^9^ Preclinical studies have demonstrated that ^161^Tb exhibits a higher relative biological effectiveness at the cellular level than ^177^Lu and can provide a superior therapeutic effect, particularly in eradicating micrometastases.^11^, ^12^

The success of RNT relies heavily on the accurate monitoring and quantitative evaluation of the in vivo biodistribution of radiopharmaceuticals. Personalized dosimetry approaches, which involve calculating absorbed radiation doses at both the organ and tumor levels, require highly accurate measurements of activity. Although images are predominantly interpreted qualitatively in current clinical practice, the quantitative analysis of radionuclide distribution is of paramount importance for treatment planning, dose optimization, and toxicity management.

Single Photon Emission Computed Tomography (SPECT), which is used for this purpose, offers significant advantages over planar imaging, including reduced organ overlap, enhanced contrast in small tumors, and more precise activity quantification in heterogeneous uptakes. However, the multiple gamma emissions of ^161^Tb (48.9 keV, 74.6 keV energy photons) can affect image quality by increasing the scatter fraction during SPECT imaging. To improve image quality, appropriate scatter correction methods must be optimized.

In nuclear medicine imaging, various scatter correction techniques are used to enhance the quality of both quantitative and qualitative images. Window‐based techniques, which are common in clinical practice, estimate scatter using additional energy windows. For example, a Dual Energy Window (DEW) uses a single scatter window, but a Triple Energy Window (TEW) computes scattering using narrow windows on either side of the photopeak window. In contrast, model‐based and Monte Carlo methods incorporate scattering as a physical model into the forward projection or iterative reconstruction process, rather than removing it after processing. Despite their widespread use in clinical practice, studies investigating the efficacy of spectral analysis approaches, such as DEW and TEW, for next‐generation radionuclides with multiple gamma emissions, such as ^161^Tb, remain limited.^6^, ^8^ The increasing use of ^161^Tb in RNT necessitates re‐optimizing scatter correction strategies.

The aim of this study is to evaluate the effects of DEW and TEW scatter correction methods on image quality and quantitative accuracy in ^161^Tb SPECT imaging. Comparative analyses were performed using parameters such as calibration factor (CF), contrast‐to‐noise ratio (CNR), signal‐to‐noise ratio (SNR), spatial resolution, and activity bias (%). The DEW/TEW scatter corrected images were compared with uncorrected (NC—No Correction) images. This study's results are expected to support the development of optimal imaging procedures for ^161^Tb‐based theragnostic applications and enable highly precise individualized dosimetry calculations.

## 2 MATERIALS AND METHODS

### 2.1 Phantom preparation and geometries

**FIGURE 1:**
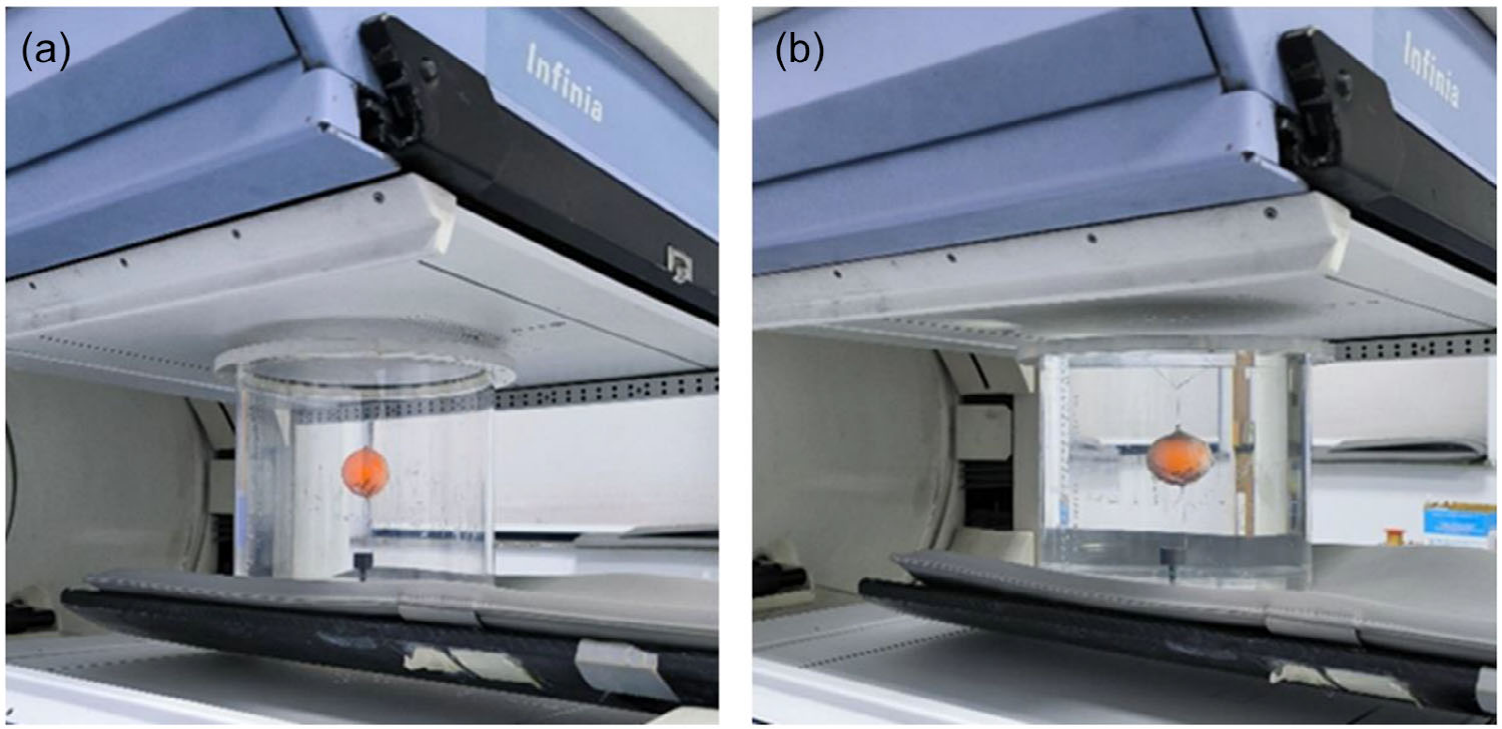
Sphere in Jaszczak phantom with (a) air and (b) water.

Three distinct reference geometries were utilized to determine the gamma camera calibration factor and to evaluate the image quality parameters. These geometries are as follows: (1) a sphere in the air (diameter of 40 mm, 31.5 mL); (2) a sphere placed inside a water‐filled Jaszczak phantom without Tb‐161; and (3) a sphere inside a Jaszczak phantom with Tb‐161 (Figure 1). Detector dead time can affect quantitative accuracy by causing actual counts to be underestimated. To mitigate these effects, the activity concentration used in this phantom setup was prepared at 7.49 MBq/mL. Tb‐161 was filled into the sphere homogeneously without any air gaps. This approach is consistent with recent guidelines recommending specific dose ranges in Tb‐161 imaging to maintain linear detector response and ensure quantitative reliability.^13^ All activities were measured using a dose calibrator (CRC‐55tR, Capintec Inc.).

### 2.2 SPECT acquisition and image reconstruction

Imaging was performed using a dual‐head (GE Infinity) gamma camera equipped with 3/8‐inch‐thick NaI(Tl) crystals and low‐energy, high‐resolution (LEHR) collimators. SPECT images of the phantom were acquired in the step‐and‐shoot mode using a 128 × 128 matrix and a zoom factor of 1.0. The total angular rotation was 360°, with 120 projections (views) acquired at 3° per step. The acquired SPECT images were reconstructed using the ordered subset expectation maximization (OSEM) algorithm with 2 iterations and 10 subsets. Following reconstruction, a Butterworth post‐filter with a cutoff frequency of 0.48 cycles/cm and an order of 10 was applied to suppress image noise. No resolution recovery was applied to isolate the effects of the scatter correction methods.

### 2.3 Scatter correction methods and energy window settings

Five image protocols were used, each with different combinations of scatter windows based on the low‐energy photopeaks of ^161^Tb (48.9 keV and 74.6 keV) (Table 1). The identification of these energy window (EW) combinations was based on previous studies.^6^

**TABLE 1:** Energy window widths of image protocols (EW1‐EW5).

Protocol name	Main photopeak	Lower scatter widths	Upper scatter widths
EW1	74.6 keV ± 10%	62.3 ± 6%	88.3 keV ± 6%
EW2	48.9 keV ± 10%	41 keV ± 6%	58 keV ± 6%
EW3	74.6 keV ± 10%	62.5 keV ± 4%	87 keV ± 4%
EW4	48.9 keV + 30% 48.9 keV – 15%	35 keV ± 14%	65 keV ± 2%
EW5	74.6 keV ± 10%	65 keV ± 2%	88 keV ± 6%

**FIGURE 2:**
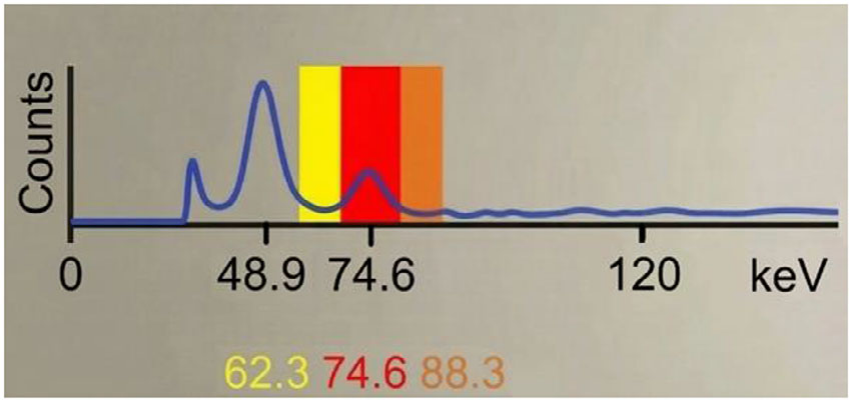
Tb‐161 spectrum with energy windows (Protocol EW1).

The DEW method, proposed by Jaszczak et al., uses a photopeak and a wide energy window positioned to the lower‐energy side of the main photopeak.^14^ Scattered photon counts are included in the counts within the main energy window. Therefore, the DEW method is based on the principle of subtracting the counts in the scatter window (C_sca_) from the total counts in the photopeak energy window (C_tot_).^15^ In the TEW approach, narrow scatter energy windows are placed on both sides of the main energy window, as illustrated in Figure 2.^16^ The scatter distribution under the main photopeak is estimated using the counts from these two scatter windows (C_left_ and C_right_). Similar to the DEW technique, a correction is applied by subtracting the estimated scatter contribution from the total counts in the main window.

(1) $$C_{prim}=C_{tot}-C_{sca}$$

(2) $$C_{sca}≅\left(\frac{C_{left}}{W_{s}}+\frac{C_{right}}{W_{s}}\right).\left(\frac{W_{m}}{2}\right)$$

In Equation 2, W_s_ and W_m_ represent the widths of the main and scatter windows, respectively, and C_prim_ represents the unscattered photon counts.^17^

### 2.4 VOI and image quality parameters

**FIGURE 3:**
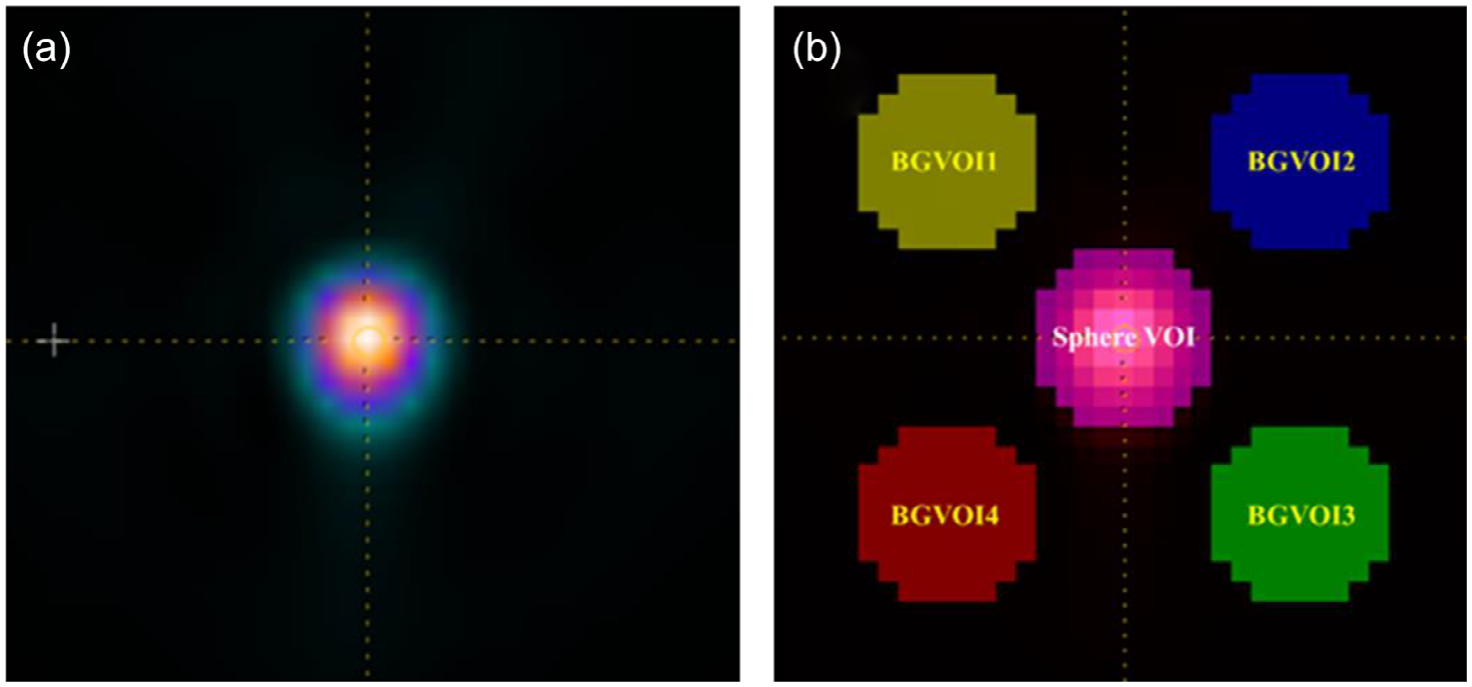
(a) Transaxial slice of Tb‐161 sphere SPECT image, (b) VOIs on that image.

Image quality parameters were calculated by drawing the volume of interest (VOIs) using LifeX software (version 25.06.1). To ensure quantitative accuracy and mitigate the boundary uncertainty caused by septal penetration, a known effect of 161Tb's higher‐energy emissions on LEHR collimators, a spherical VOI was utilized for the source. Specifically, one VOI was defined for the sphere, matching its physical inner diameter (40 mm), and four identical VOIs were placed in the background regions as shown in Figure 3. The CF, CNR, SNR, spatial resolution (full‐width at half maximum, FWHM) and activity bias (%) data were compared in both uncorrected and corrected SPECT images.

#### 2.4.1 System sensitivity and calibration factor

Initially, after performing energy calibration with a Tc‐99 m point source according to clinical routine, a homogeneity map was generated using Tb‐161. A flat circular petri dish with a diameter of 15 cm was used to determine the optimal imaging settings and energy windows. It was placed between the detectors, approximately 5 cm from each detector surface. For imaging photon emissions from Tb‐161, gamma emission windows of 48.9 keV ± 10% and 74.6 keV ± 10% were used as the lowest and highest, respectively. A static image was acquired in a 256 × 256 matrix for the LEHR collimator. Five million counts were collected for both detectors.

The CF, which is used to convert SPECT image counts to absolute activity concentrations, was calculated using Equation 3.^18^, ^19^

(3) $$CF=\frac{Count}{A\times t}\left(\frac{cps}{MBq}\right)$$

In Equation 3, ‘Count’ corresponds to the total counts acquired from each source VOI; ‘A’ represents the activity in the sphere; and ‘t’ defines the acquisition time. Calibration factors for images, which were taken at three different reference geometries, were calculated using decay‐corrected activity values ​​based on the physical half‐life of Tb‐161.

#### 2.4.2 Contrast‐noise ratio and signal‐noise ratio

CNR is a measure of the contrast between the target and background; this parameter characterizes the distinguishability and detectability of lesions, particularly those with low contrast, within the background.^20^ The SNR determines the statistical quality and clarity of an image. It is the ratio between the average signal intensity in the region of interest and the background noise.^21^, ^22^ The CNR and SNR were calculated using the following equations:

(4) $$CNR=\frac{\left|C_{s}-C_{bg}\right|}{\sigma_{bg}}$$

(5) $$SNR=\frac{C_{s}}{\sigma_{s}}$$

In the equations, $C_{s}$ and $C_{bg}$ correspond to the mean counts in the ROIs defined for the source and background (BG), respectively, while $\sigma_{s}$ and $\sigma_{bg}$ represent the standard deviation values in the ROIs defined for the source and BG.

#### 2.4.3 Spatial resolution

Profile curves of NC and DEW/TEW corrected images were generated using an Xeleris workstation, and the FWHM data were obtained from these curves to determine the spatial resolution, which indicates how well two adjacent point sources in the image can be distinguished.

#### 2.4.4 Activity bias

The activity bias, which evaluates absolute quantification accuracy and the impact of septal penetration from the LEHR collimator, was calculated. It quantifies the percentage of overestimation or underestimation relative to the known true activity. The bias was calculated using the following equation:

(6) $$Bias\left(\%\right)=\left(\frac{C_{measured}-C_{true}}{C_{true}}\right)\times 100$$

where C_measured_ is the activity concentration derived from the SPECT images using the system calibration factor, and C_true_ is the known activity concentration.

## 3 RESULTS

The impact of optimal energy window selection and scatter correction methods on Tb‐161 SPECT imaging was evaluated using system sensitivity, visual analysis, and quantitative image quality parameters.

### 3.1 System sensitivity and calibration factors

Sensitivity measurements were calculated separately for both detectors. For 74.6 keV ± 10%, the per‐detector sensitivity values for Detector 1 and Detector 2 were 8.8 cps/MBq and 7.62 cps/MBq, respectively. For the dual‐window configuration (combining both the 48.9 keV ± 10% and 74.6 keV ± 10% energy windows), the per‐detector sensitivities were 22.11 cps/MBq and 21.37 cps/MBq, respectively.

**FIGURE 4:**
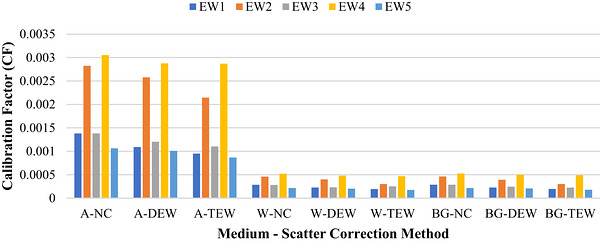
Change in CF due to medium and scatter correction in Tb‐161 SPECT images. A, air; W, water; BG, background; NC, no correction; DEW, dual‐energy window; TEW, triple‐energy window.

When comparing CF values across all geometries, higher CF values were calculated for the NC images, while the lowest CF values were found in the TEW‐corrected images (Figure 4). Among the calibration factors of the gamma camera system, the highest value was calculated for images taken in air. In the water medium, significant attenuation was observed due to the low‐energy gamma rays from Tb‐161. However, when comparing both photopeaks of Tb‐161, the CF calculated in images acquired with an energy window of 48.9 keV was found to be higher than that calculated in an energy window of 74.6 keV. In images acquired in the medium with background activity, the CF values calculated after scatter correction are consistent with the values in the water medium.

### 3.2 Impact of DEW and TEW scatter correction methods

The DEW and TEW scatter correction techniques improve image clarity in images acquired using three distinct media. Edge sharpness was more noticeable in images acquired using the TEW method than the DEW method. In addition, scatter correction techniques reduce background noise in images acquired in water with and without background activity, where scattering is considerable.

### 3.3 Quantitative evaluation and image quality parameters

#### 3.3.1 Contrast to noise ratio

**FIGURE 5:**
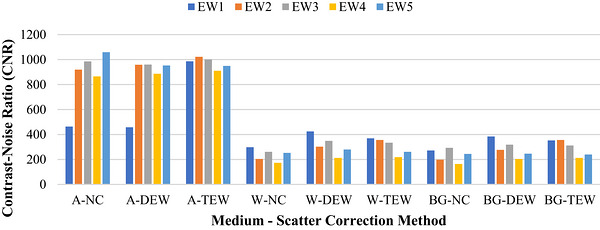
Change in CNR due to medium and scatter correction in Tb‐161 SPECT images. A, air; W, water; BG, background; NC, no correction; DEW, dual‐energy window; TEW, triple‐energy window.

The CNR parameter in Tb‐161 SPECT imaging varied with the physical characteristics of the medium and applied scatter correction techniques (Figure 5). The best contrast was obtained with the EW2 protocol in air, whereas in scattered media, it was observed in images obtained with the EW1 protocol. The highest CNR was calculated to be 1022.2 in the TEW‐corrected image in air, 356.4 in the TEW‐corrected image in water, and 384.7 in the DEW‐corrected image in the active background medium.

#### 3.3.2 Signal to noise ratio

**FIGURE 6:**
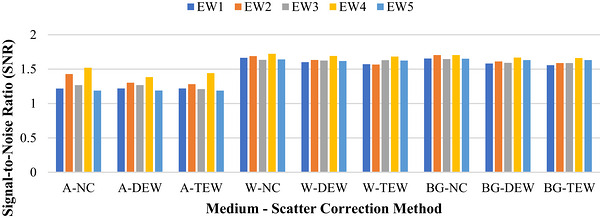
Change in SNR due to medium and scatter correction in Tb‐161 SPECT images. A, air; W, water; BG, background; NC, no correction; DEW, dual‐energy window; TEW, triple‐energy window.

Figure 6 presents the SNR values calculated from the SPECT images acquired in the three different media. When scatter correction (DEW and TEW) was applied, the images’ SNR values decreased slightly, ranging from 1.188 to 1.724 across all media. When comparing the media, the SNR values in the scattering medium were slightly higher than those in the air medium.

#### 3.3.3 Spatial resolution

**FIGURE 7:**
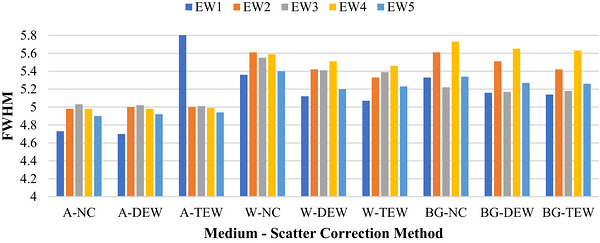
Change in FWHM due to medium and scatter correction in Tb‐161 SPECT images. A, air; W, water; BG, background; NC, no correction; DEW, dual‐energy window; TEW, triple‐energy window.

The FWHM values representing spatial resolution in Tb‐161 SPECT imaging quantitatively demonstrated the impact of scatter‐correcting methods on edge sharpness. In water with and without activity media, the NC images provided the lowest resolution with the highest FWHM values. Scatter correction techniques reduced the FWHM values for all protocols except the air medium, thereby improving spatial resolution. Using the TEW approach, the FWHM values in water with or without activity were 5.07 and 5.14 mm, respectively. When the profile curves in Figure 7 are examined, it is observed that the scatter tails of the curves associated with the corrected images are flattened.

#### 3.3.4 Activity bias

According to the calculated activity bias values, the activity concentration in NC images was overestimated. Although scatter corrections reduced this overestimation, the activity bias after correction remained high for this low‐energy peak; the TEW correction method reduced the calculated activity bias in NC images by 63.4% in water and 61.7% in the active background, but the absolute biases remained at 43.4% and 49.1%, respectively.

In contrast, the 74.6 keV photopeak (EW1 protocol) provided quantitative accuracy across all media. The DEW correction method reduced the activity bias of these NC images by 70.4% in water and 69.9% in active background, lowering the absolute biases to 12.0% and 12.4%, respectively.

The TEW approach effectively reduced scattering even in active background for the 74.6 keV peak. It reduced the calculated activity bias in NC images by 89.7% in water and 91.0% in active background, lowering the absolute activity biases to 4.2% and 3.7%, respectively. In images acquired in air, the TEW method reduced activity bias by 36.6% compared to other methods. These findings demonstrate that TEW correction can be applied to ensure absolute accuracy in scattering media in Tb‐161 imaging acquired with a 74.6 keV photopeak.

## 4 DISCUSSION

Scattered photons, due to the Compton effect within the tissue, change direction and energy before reaching the detector, thereby affecting signal accuracy and leading to contrast loss, reduced spatial resolution, and quantitative errors. Low‐energy photons are more likely to scatter, which increases the proportion of scattered photons that fall within the photopeak window. It is widely acknowledged that Compton scattering is the primary interaction in tissues, although the photoelectric effect is the primary mechanism of photon interaction in detectors. Coherent scattering also occurs at energies below 150 keV; however, because of the narrow deflection angle, coherently scattered photons are similar to the primary photons.^17^, ^23^

It has been reported that the scatter contribution in NC SPECT images can reach up to 30% of the total counts.^24^, ^25^ Therefore, problems such as scatter and attenuation must be corrected using appropriate techniques to improve image quality and enable precise quantitative evaluations.^4^, ^26^, ^27^, ^28^, ^29^ Among the window‐based scatter correction methods, DEW and TEW are readily applied to SPECT images.^17^, ^30^, ^31^ In this study, the effects of window‐based scatter correction methods on image quality and quantitative accuracy in Tb‐161 SPECT imaging were systematically evaluated. The findings demonstrated that both scatter correction methods increased the CNR and improved spatial resolution, while resulting in a slight, predictable decrease in SNR.

In images acquired using the 48.9 keV photopeak window of ^161^Tb, the collected signals are degraded by low energy resolution, and this photopeak window is affected by photons scattered from the 74.6 keV photopeak. Therefore, performing scatter correction in the 48.9 keV photopeak energy window is quite difficult. In the phantom study by Marin et al. using Tb‐161, it was emphasized that images acquired at the 48.9 keV photopeak showed lower image quality than those acquired at the 74.6 keV photopeak. Therefore, the authors recommended using the highest‐energy gamma emission in imaging.^6^

In their study using the I‐123 radionuclide, Michael et al. reported that, in the absence of background activity, the counts in NC and scatter‐corrected images showed a linear relationship.^30^ Furthermore, they found that the TEW method outperformed the DEW method in both quantitative analysis and qualitative evaluation, particularly in environments with high background activity.

The high abundance of Tb‐161 characteristic X‐rays in this energy range and the contribution of down‐scattered higher‐energy photons into this window due to Compton scattering within the phantom contribute to the high‐count rates observed in the 48.9 keV photopeak window in our study.^6^, ^12^, ^21^

The factor affecting quantitative accuracy in Tb‐161 SPECT imaging is detector dead time. The choice of applied dose or phantom activity must be carefully optimized, as increased counting rates due to scattering associated with Tb‐161 can lead to significant dead time and consequently an underestimation of true activity. For clinical applications and future phantom studies using LEHR collimators, adherence to practical dose ranges that avoid significant dead time effects is strongly recommended, as detailed in the study by Westerbergh et al.^13^

SPECT imaging can be performed using therapeutic amounts of Tb‐161, and high image quality can be achieved with a 74.6 keV ± 10% energy window. The LEHR collimator is a suitable choice for ensuring high resolution in the acquired images. Therefore, dosimetry using Tb‐161‐labeled radiopharmaceuticals is feasible in clinical settings.

### 4.1 Effects on CNR

The results of this study show that, in comparison with NC images, both DEW and TEW scatter correction techniques considerably increase CNR values, which is in line with previous research.^8^, ^32^, ^33^, ^34^ The TEW method improves image contrast, according to a simulation study by Noori‐Asl et al. assessing six scatter correction techniques.^35^ The highest CNR value was obtained when TEW correction was applied to images acquired under the EW2 protocol, with and without background activity. These quantitative results show that TEW is usually better than DEW in regard to contrast, and the choice of spectral method parameters can dramatically change the resulting CNR, even though model/Monte Carlo‐based approaches yield the most noticeable improvements in contrast and CNR.^32^, ^34^

The lower CNR obtained in images acquired in the scattering medium compared to the air medium is due to the high scattering and absorption of low‐energy Tb‐161 photons within the phantom containing the scattering medium. In images acquired in water and active‐background media, both scatter‐correction methods improved contrast compared to the NC images.

### 4.2 Effects on SNR

A decrease in total counting statistics due to scatter correction methods usually reduces the SNR. The SNR values for scatter‐corrected images in our study varied from 1.188 to 1.724, with NC images showing the highest values. This is a direct result of removing scattered photons and is in accordance with the literature.^36^, ^37^, ^38^ Rafati et al. also found that TEW improves image contrast while reducing the SNR.^34^

### 4.3 Effects on spatial resolution

Improving spatial resolution is critical for increasing diagnostic accuracy in SPECT imaging. The FWHM values in our study ranged from 4.7 to 5.73 mm across all media. According to the study, the spatial resolution in the scattering medium was improved using the DEW and TEW scatter correction methods, resulting in more distinct object boundaries. This enhancement demonstrates how scatter correction reduces blurring effects on the image, thereby improving spatial resolution. Tantawy et al. reported that scatter correction techniques enhanced spatial resolution in a study comparing four correction methods for myocardial perfusion SPECT/CT images.^31^

### 4.4 Effects on activity bias

The activity bias results highlight the impact of scatter and septal penetration on Tb‐161 quantification. The overestimation observed in NC protocols using the 48.9 keV photopeak suggests that the primary signal in this low‐energy window is likely affected by down‐scattered photons and penetration artifacts. In contrast, assessments using the 74.6 keV peak (EW1 protocol) showed a much lower bias without correction, which was improved by scatter correction methods. Specifically, the TEW correction method reduced the calculated activity bias in NC images by 89.7% to 91.0% in scattering media. This improvement resulted in an activity bias of 4.2% in water and 3.7% under active background conditions. This quantitatively demonstrates that the 74.6 keV window is important for minimizing activity overestimation and improving overall dosimetric accuracy.

## 5 LIMITATIONS REGARDING ATTENUATION CORRECTION

A notable limitation of this study is the absence of attenuation correction (AC), specifically computed tomography‐based attenuation correction (CTAC). Our primary focus was isolating the efficacy of window‐based scatter correction methods. However, the low‐energy emissions of Tb‐161 are highly susceptible to tissue attenuation, which exerts a profound effect on absolute quantification accuracy, as highlighted by recent literature.^8^, ^13^ In this context, the application of analytical Chang's AC method was technically precluded because the automated boundary detection algorithms within the processing software failed to accurately delineate the outer physical contours of the phantom, thereby preventing the generation of the requisite attenuation map. Ultimately, while our optimized TEW protocol effectively mitigates scatter, true patient‐specific dosimetry in heterogeneous clinical anatomy mandates the integration of these optimized energy windows with hybrid SPECT/CT AC to fully compensate for attenuation losses.

A further limitation of our quantitative analysis is the use of a VOI matched to the 40 mm physical diameter of the spherical source. This approach inherently mixes partial‐volume effects, septal penetration, and residual scatter. While this is acceptable for the relative methodological comparisons performed in this study, future investigations evaluating absolute quantification could consider using a smaller central VOI to isolate scatter effects more precisely.

## 6 CONCLUSION

In this study, the impact of scatter correction methods on SPECT images of the recently developed radiopharmaceutical ^161^Tb was assessed qualitatively and quantitatively. Our findings indicate that both DEW and TEW correction methods improve spatial resolution by sharpening object boundaries and increase CNR by reducing scattering contributions, although with a slight reduction in SNR due to decreased total counting statistics. The TEW approach outperformed the DEW approach in terms of quantitative accuracy under complicated scattering conditions (water with/without activity). In SPECT imaging using therapeutic amounts of Tb‐161, high image quality can be achieved with an energy window of 74.6 keV ± 10%.

Based on our quantitative results, we highly recommend utilizing the 74.6 keV photopeak (± 10%) in conjunction with the TEW scatter correction method (lower scatter window: 62.3 keV ± 6%, upper scatter window: 88.3 keV ± 6%). When combined with standard LEHR collimators and OSEM reconstruction, this specific configuration (our EW1 protocol) optimally balances spatial resolution, contrast, and quantitative accuracy for ^161^Tb imaging.

Although this research was limited to standard phantom geometries, it confirms that window‐based techniques can be used in ^161^Tb SPECT imaging. Further in vivo research that considers complex anatomical backgrounds and patient physiology is needed to establish accurate clinical dosimetry protocols. Additionally, the scope of this study was limited to clinically accessible window‐based methods (DEW/TEW) without a direct performance comparison with advanced techniques, such as Monte Carlo‐based reconstruction models.

## AUTHOR CONTRIBUTIONS

All authors contributed to the material preparation, data collection, and analysis. The first draft of the manuscript was written by Can M. And all authors commented on previous versions of the manuscript. All authors read and approved the final manuscript.

## CONFLICT OF INTEREST STATEMENT

The authors declare no conflicts of interest.

## Data Availability

The datasets are available from the corresponding author on reasonable request.
