# Correction to “Improving image quality in terbium‐161 phantom imaging: Quantitative evaluation of DEW and TEW scatter correction methods”

**DOI:** 10.1002/acm2.70762

**Published:** 2026-09-16

Can M, Çapa Kaya G, Gümüşer G, Parlak Y. Improving image quality in terbium‐161 phantom imaging: Quantitative evaluation of DEW and TEW scatter correction methods. *J Appl Clin Med Phys*. 2026;e70672. https://doi.org/10.1002/acm2.70672

In the original publication, a reference for the LIFEx software was inadvertently omitted in the Materials and Methods section.

Under the subheading “**2.4. VOI and Image Quality Parameters**”, the first sentence should be corrected to include the relevant software citation as follows:

Image quality parameters were calculated by drawing the volume of interest (VOIs) using LifeX software (version 25.06.1).^18^

**Reference to be added**: Nioche C, Orlhac F, Boughdad S, et al. Lifex: A freeware for radiomic feature calculation in multimodality imaging to accelerate advances in the characterization of tumor heterogeneity. *Cancer Res*. 2018;78(16):4786‐4789. doi:10.1158/0008‐5472.CAN‐18‐0125

